# Assessing the quality of educational short videos on dry eye care: a cross-sectional study

**DOI:** 10.3389/fpubh.2025.1542278

**Published:** 2025-04-09

**Authors:** Mingxue Huang, Jiaoman Wang, Jiawen Wei, Rongkui Zhang, Xiaoyan Wang, Jinhua Gan, Zhe Zhang, Fangyan Liu

**Affiliations:** ^1^School of Nursing, Southwest Medical University, Luzhou, China; ^2^Shenzhen Eye Hospital, Shenzhen Eye Medical Center, Southern Medical University, Shenzhen, China; ^3^Affiliated Hospital of Southwest Medical University, Luzhou, China

**Keywords:** dry eye, care, short video, social media, quality, education

## Abstract

**Background:**

Short video social media platforms play a crucial role in public health by effectively disseminating health information. Despite this, many educational videos on dry eye care have not received sufficient attention. This study aimed to conduct a comprehensive analysis and evaluate the quality of educational short videos on dry eye care available on TikTok.

**Methods:**

On August 30, 2024, the top 200 videos related to dry eye were viewed from the Chinese version of TikTok using the platform’s default ranking. The overall quality, reliability, comprehensibility, and applicability of the videos are systematically evaluated using the DISCERN and PEMAT-A/V assessment tools.

**Results:**

A total of 199 videos were included in the study and categorized based on account information: medical professional individual users, general professional individual users, for-profit organizations, non-profit organizations, and news organizations. Medical professionals were the predominant uploaders, contributing 81% of the videos. The overall misinformation rate was 2%. A majority of the videos (85.9%) addressed at least two aspects of dry eye, while only 14.1% covered three or more topics. The videos scored 22.4 ± 6.4 for reliability and 17.4 ± 6.2 for treatment options. Upon evaluation, the understandability and actionability of these videos were found to be 79.1% and 60.4%, respectively.

**Conclusion:**

TikTok holds significant potential for disseminating health information, primarily through content created by medical professionals. Currently, much of the content focuses on the symptoms and management of dry eye, with limited discussion on its definition, classification, and diagnosis. While most video content is reliable, there is a risk of incomplete or inaccurate information, these videos can serve as a reference. Therefore, the public should exercise caution when seeking information on dry eye through TikTok and individuals experiencing symptoms are advised to consult healthcare professionals for accurate diagnosis and treatment.

## 1 Introduction

Dry eye (DE) is a chronic ocular surface disease influenced by multiple factors, resulting in a spectrum of eye symptoms and visual impairment (1, 2). Available research indicates that the prevalence of DE, diagnosed based on clinical symptoms and signs, varies from 9% to 30% (3). Throughout the COVID-19 pandemic, this prevalence increased, with questionnaire-based assessments reporting symptomatic dry eye prevalence among students ranging from 44.1% to 92.8% (4–7). As an ocular surface disease, DE can cause symptoms such as pain, redness, and visual disturbances, significantly disrupting daily activities like reading, writing, or working on video screens. This disruption adversely affects an individual’s eye health, quality of life, and overall sense of well-being (8–10). Therefore, it is crucial to acknowledge and address the issue of DE.

Social media is extensively utilized worldwide, providing a platform for promoting public health and disseminating health information (11). During the COVID-19 pandemic, internet platforms, including short video formats, have played a vital role in disseminating health information (12, 13). More than 70% of individuals are active on at least one social media platform 14. Since 2020, the user base of the short video platform TikTok has expanded rapidly, with its downloads surpassing 2 billion by August of that year (12, 14). Social media enables the public to engage in extensive discussions about health issues, free from the constraints of time and location (14, 15). Medical organizations and individuals use social media to expand their influence and to share and discuss various interests and key issues (14, 16). Social media offers patients many chances to acquire health information, empowering them to actively manage their health (17).

While social media plays a significant role in enhancing the dissemination of scientific findings, it also allows patients with similar medical conditions and healthcare professionals to share their experiences and opinions online. This can lead to a mixed quality of information, particularly in short video content, underscoring the need for monitoring the quality and reliability of shared information (18, 19). It is crucial to conduct quality reviews of the information being disseminated (15, 20). For instance, during the public health crisis, there was a notable increase in the promotion of unverified and potentially harmful COVID-19 treatments (21–23). TikTok has a large user base and has a huge reach, with many people signing up on multiple platforms to share the same video content (13, 14). Therefore, to avoid repeating the study, this study focused on TikTok as a platform. The Chinese version of TikTok contains numerous educational videos about dry eye care; however, the quality of these videos has not been systematically evaluated. The aim of this study is to conduct a systematic evaluation of educational videos related to DE on TikTok.

## 2 Methods

### 2.1 Search strategy and inclusion/exclusion criteria

On August 30, 2024, enter the keyword “干眼(Dry Eye)” on the Chinese version of TikTok (version 31.2.0). Screen the top 200 videos selected based on TikTok’s default sorting (comprehensive ranking). Exclusion criteria: (i) Videos not related to DE; (ii) Videos not in Chinese; (iii) Duplicate content; (iv) Non-original content; (v) Incomplete account information of the publisher.

### 2.2 Data extraction

Extract the main baseline characteristics of each video, including the uploader’s identity, posted dates, video length, and the number of likes, comments, shares, and saves received, as well as the video source. From August 30 to September 1, two team members collaboratively reviewed the videos and manually entered the relevant features into Excel. The videos were categorized into two groups based on the account name registered by the video publisher on the short video platform and their authentication status: individual users and organizational users. The individual users group includes medical and non-medical individual users, while the organizational users group comprises for-profit organizations (e.g., private sector organizations or advertising and marketing stores), non-profit organizations (e.g., hospital institutions, governmental accounts) and news agencies.

### 2.3 Assessment methodology

To verify the completeness and accuracy of the video content, the study uses the dry eye diagnosis and treatment consensus and the DE classification consensus published by the Asian Dry Eye Society as criteria for evaluation (1, 24). The study evaluated two aspects of dry eye-related videos on TikTok: informational content and quality. Two researchers with in-depth knowledge of dry eye reviewed the top 10 ranked videos, examined their main content, and adopted the framework suggested by Goobie et al. (25). The evaluation encompassed the following six aspects of the videos: definition of DE, classification, symptoms, risk factors, diagnosis, and treatment management.

The validated DISCERN and PEMAT-A/V tools were utilized to evaluate the quality of the videos (26–28). DICERN consists of 16 items categorized into three sections, reliability of information (items 1–8), treatment options (items 9–15), an overall quality score is assigned (item 16), with each item evaluated on a scale ranging from 1 to 5. where 1 indicates failure to meet the criteria and 5 indicates full compliance with the criteria. PEMAT-A/V consists of 17 items categorized into two sections: understandability (13 items) and actionability (4 items). Each section is scored independently. All items provide options for “disagree” or “agree,” with some items also including a “not applicable” choice. Scoring was conducted independently by two ophthalmology practitioners, each possessing a comprehensive understanding of the evaluation criteria and the guidelines for utilizing the evaluation tools. Furthermore, after both raters have assessed the video using the DISCERN tool, a reliability test will be conducted to evaluate the consistency between their ratings.

### 2.4 Statistical analysis

Data analysis is performed using IBM SPSS Statistics version 27.0. Normally distributed data are reported as the mean with the standard deviation, while categorical data are expressed as frequencies and percentages. The intraclass correlation coefficient (ICC) was computed to assess the consistency of total DISCERN scores between the two raters. A one-way analysis of variance (ANOVA) was conducted to test for score differences among various video sources. Pearson or Spearman rank correlation was utilized to explore associations between general video characteristics and assessment tool scores. A *p*-value of less than 0.05 was deemed indicative of a significant difference.

## 3 Results

### 3.1 Basic information characteristics

**Figure 1:**
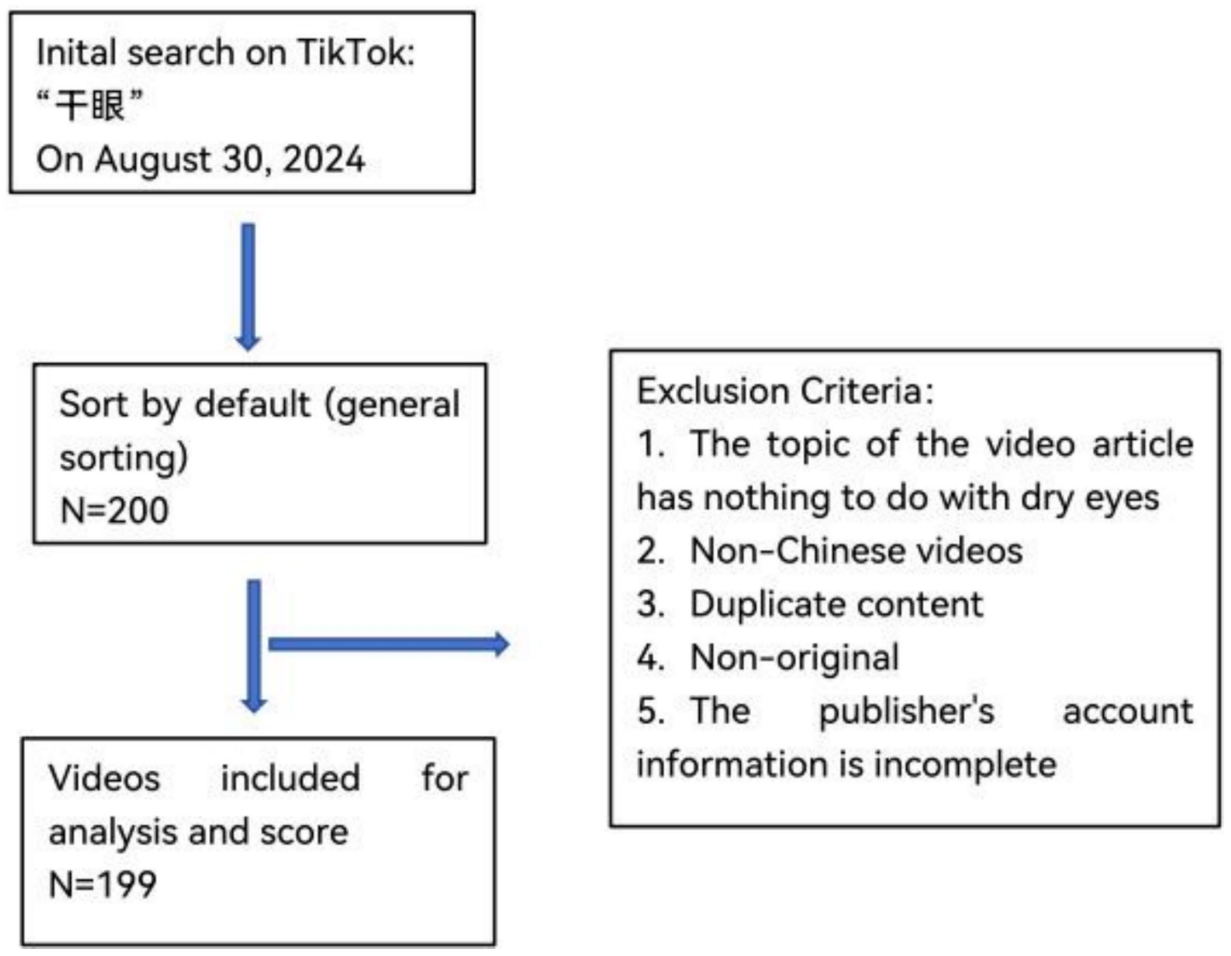
Video screening criteria and process.

**Figure 2:**
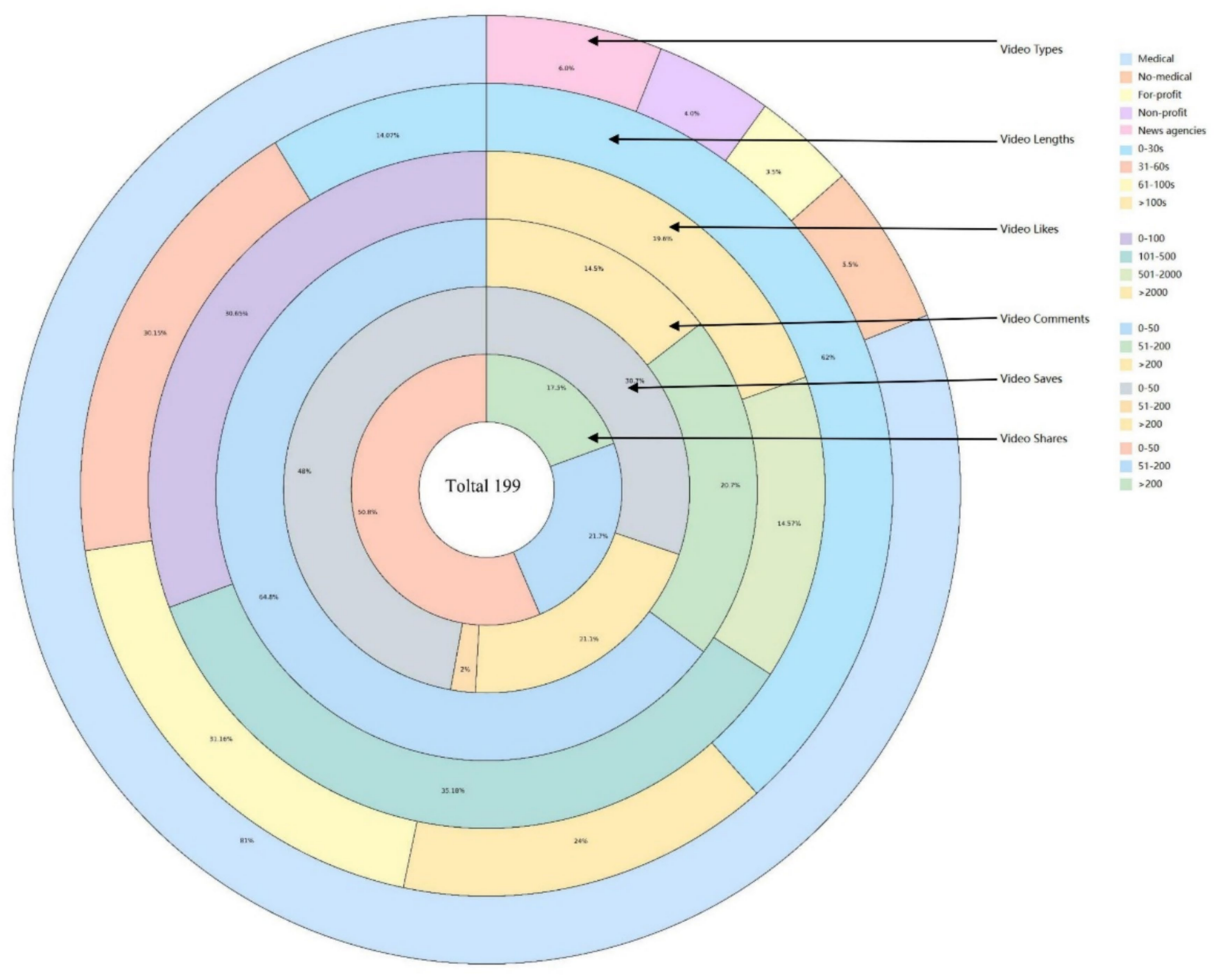
Distribution of videos in terms of types, length, likes, comments, saves, and shares. Video length: mainly concentrated in 31–60 s (30.15%) and 61–100 s (31.16%). Video likes: Most of them are below 100 (76.38%). Video comments: Less than 50 accounted for the largest proportion (64.8%). Video saves: The number is concentrated below 500 (80.4%). Video shares: The largest number of retweets is less than 50 (50.8%).

After excluding one video unrelated to the topic of DE, a total of 199 videos were included in this study (see Figures 1, 2). All video accounts are platform-certified, with the majority uploaded by medical professionals (161/199, 81%), while the fewest were uploaded by for-profit organizations (7/199, 3.5%). Collectively, the videos received 454,863 likes, 40,745 comments, 129,177 shares, and 255,726 forwards over a span of 378 days (128,640 days post-publication). The median length of the videos was 64 s, with those uploaded by for-profit organizations being the shortest at just 22 s. Videos uploaded by news agencies and non-medical individual accounts garnered higher numbers of likes, comments, saves, and shares. The specific values can be found in Table 1.

**Table 1:** Basic characteristics of the video.

Source of videos	Subgroup	*N* (%)	Days posted M(P_25_,P_75_)	Length M(P_25_,P_75_)	Likes M(P_25_,P_75_)	Comments M(P_25_,P_75_)	Saves M(P_25_,P_75_)	Shares M(P_25_,P_75_)
Individual user	Medical	161 (81)	380(127,650)	64(40,94)	227(94,975)	23(8,98)	62(21,335)	46(14,290)
	Non-medical	11 (5.5)	387(248,640)	93(63,262)	299(101,825)	85(53,372)	88(30,348)	98(26,355)
Organizational user	For-profit	7 (3.5)	38(7,216)	22(16,45)	5(3,188)	1(0,22)	33(1,62)	2(1,86)
	Non-profit	8 (4.0)	174(87,532)	67(0,117)	72(23,128)	11(1,16)	14(5,39)	9(5,44)
	News agencies	12 (6.0)	522(143,1,450)	103(38,268)	543(52,9,502)	16(3,212)	740(13,2,949)	844(26,2030)
Overall		199 (100)	378(128,640)	64(40,100)	199(87,929)	22(8,101)	53(19,340)	48(12,292)

M, Median. (P_25_,P_75_) represented interquartile range. *N*, number.

### 3.2 Video content

**Figure 3:**
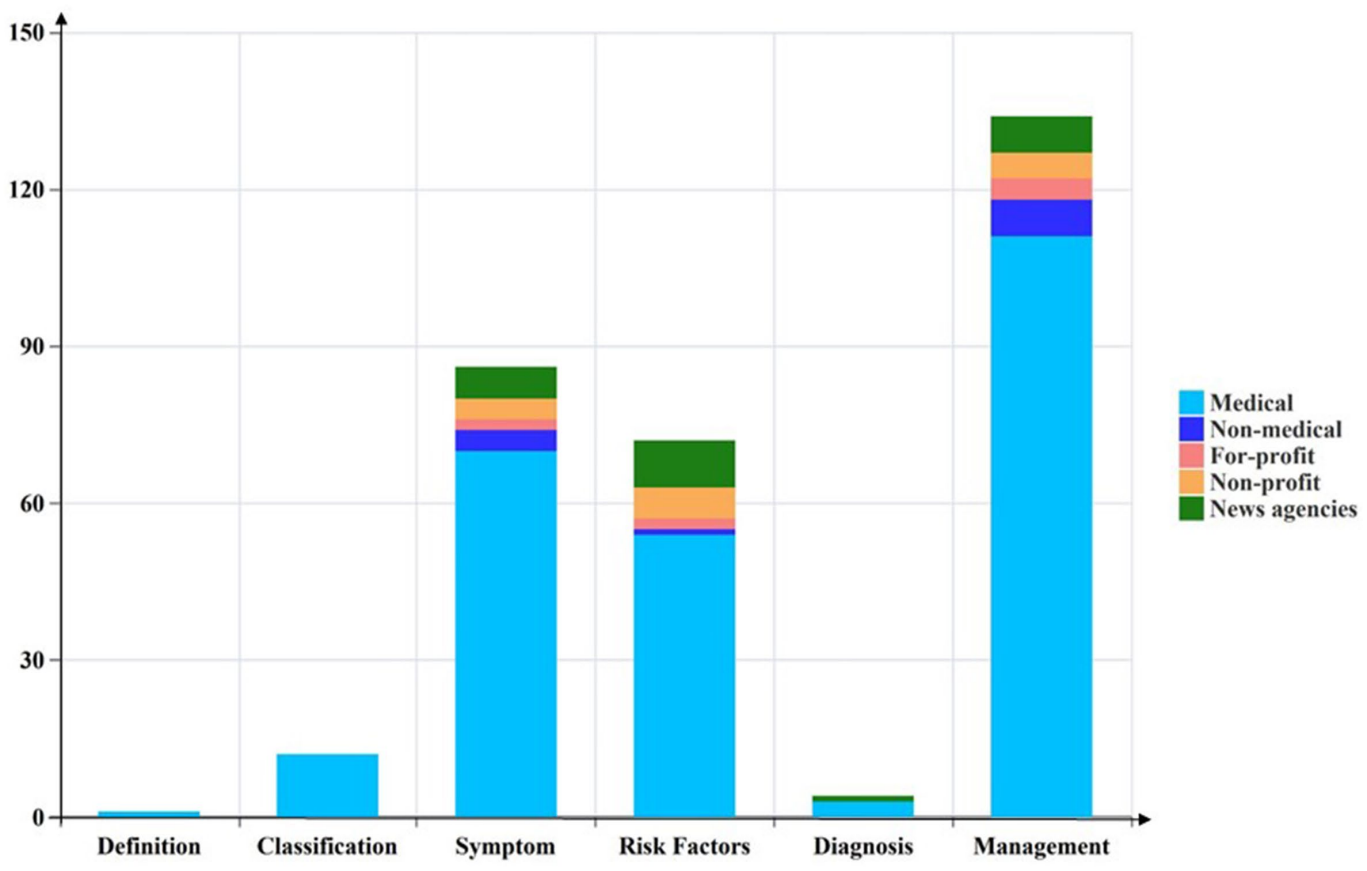
The videos cover various aspects of DE.

In the study, 85.9% of the included videos mentioned at least two out of six content topics. However, only 14.1% of the videos addressed three or more of these topics. Specifically, 63.7% of the videos discussed strategies for managing dry eye symptoms, while 43.2% and 36.1% addressed the symptoms themselves and associated risk factors, respectively. Videos that simultaneously covered the definition, diagnosis, and classification of dry eye represented less than 10% of the total. Among videos uploaded by medical professionals, only one referenced the definition of DE (see Figure 3).

### 3.3 Misinformation

Among the 199 videos included in the analysis, 12 were uploaded by individual accounts of medical professionals and addressed the classification of DE. Four of these videos were deemed incorrect due to misclassification of dry eye types, resulting in an error rate of 2% (4/199). These videos primarily classified dry eye into aqueous-deficient and evaporative types.

### 3.4 Information quality

**Figure 4:**
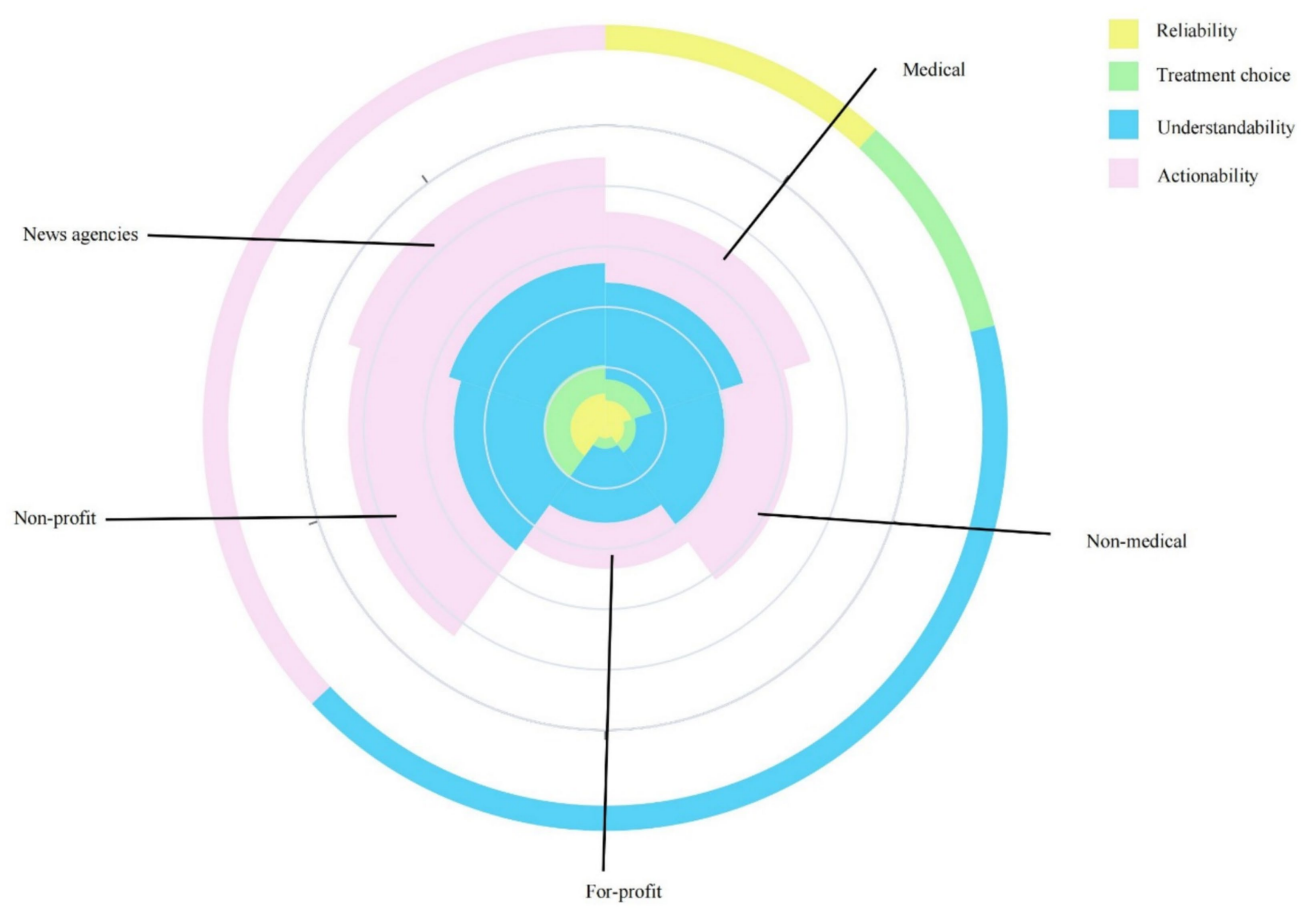
Comparison of the quality of the videos from five sources.

In this study, the reliability of videos was assessed using items 1–8 of the DISCERN tool, resulting in an average reliability score of 22.4 ± 6.4. Videos produced by news organizations demonstrated higher reliability compared to those by medical professionals (*p* = 0.004), and were also rated higher than those by non-medical individuals and commercial organizations (*p* < 0.001). Furthermore, videos uploaded by medical professionals were found to be more reliable than those from non-medical individuals and commercial entities (*p* < 0.001). Items 9–15 of the DISCERN tool were used to evaluate the quality of information for patients making treatment decisions, yielding an average score of 17.4 ± 6.2 for the videos included in this research. News agencies and non-profit organizations attained higher scores, despite the absence of a significant difference between non-profit organizations and medical professionals (*p* = 0.054). The overall quality of each video was evaluated using the 16th item of the DISCERN tool, resulting in an overall score of 3. News organizations exhibited higher overall quality than non-medical individuals and for-profit organizations (*p* < 0.001). The consistency between the two raters for the DISCERN total score was 0.85 (*p* < 0.001). The understandability and actionability of the sample were assessed using the PEMAT-A/V tool, yielding scores of 79.1% and 60.4%, respectively. The actionability of news organizations (86.1%) was higher than that of medical professionals (86.1% vs. 58.5%, *p* = 0.024) (see Figure 4). The specific values can be found in Table 2.

**Table 2:** Scores in various aspects of the videos.

Variables	Overall (*n* = 199)	Individual users		Organization users			*p* value
		Medical (*n* = 161)	Non-medical (*n* = 11)	For-profit (*n* = 7)	Non-profit (*n* = 8)	News agencies (*n* = 12)	
Videos reliability(*n* = 199)	22.4 ± 6.4	22(19,26.5)	15.6 ± 5.1	6(5,8)	28.8 ± 5.8	28.4 ± 6.8	<0.001
Treatment choice(*n* = 199)	17.4 ± 6.2	18(13.5,20)	9.7(2.6)	5(6,7)	22.6 ± 5.8	18(24,28.8)	<0.001
DISCERN tool scores	42.5 ± 11.8	36(43,50)	27.2 ± 7.5	11(13,16)	54.4 ± 11.2	60(45.5,65.3)	<0.001
Overall quality score (*n* = 199)	3.0(2,3)	2.9 ± 0.8	2.1 ± 0.8	1(1,2)	3.0 ± 0.8	3.4 ± 1.0	<0.001
PEMAT-A/V understandability	80%(72.7,88.8%)	80.1% ± 7.8%	73.3% ± 9.0%	61.1% ± 15.3%	74.3% ± 8.4%	84.45% ± 7.5%	<0.001
PEMAT-A/V actionability	60.3%(33.3,66.7%)	58.5%(31.3%)	62.1%(33.3,66.7%)	33.% ± 23.0%	83.3%(66.7,87.8%)	86.1% ± 26.4%	<0.001
Reported 1–2 contents(%)	171 (85.9%)	137(68.8%)	11(5.5%)	7(3.5%)	6(3.0%)	10(5.0%)	
Reported 3–4 contents, *n*(%)	26 (13.1%)	20(10.1%)	0	0	2(1.0%)	2(1.0%)	
Reported 5–6 contents (%)	2 (1.0%)	0	0	0	0	2(1.0%)	

*P* represented the overall significance of the difference in five subgroups. M, Median. (P_25_,P_75_) represented interquartile range. *N*, number.

### 3.5 Correlation analysis

**Figure 5:**
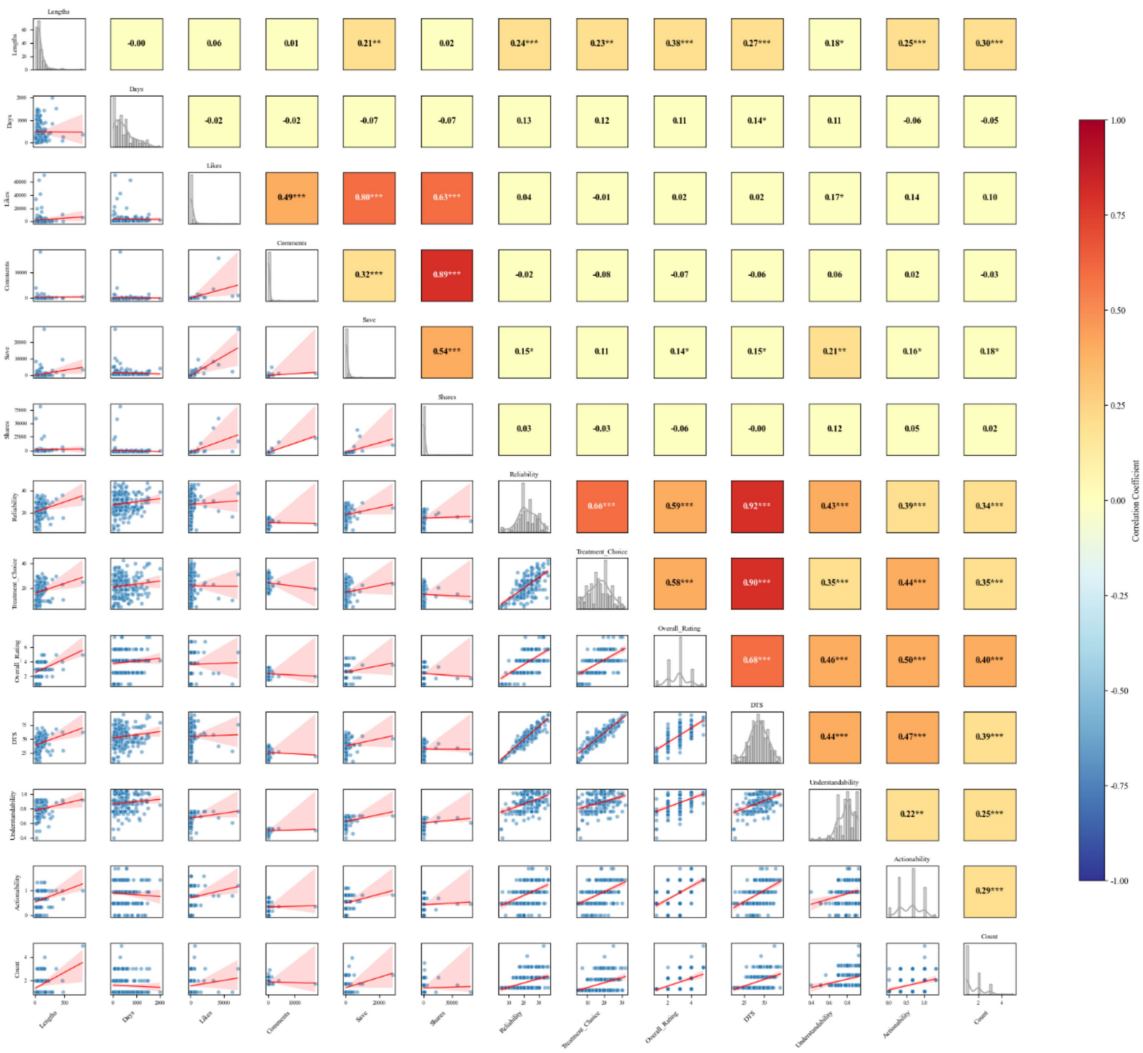
Video features correlation heatmap. DST, DISCERN total score. Count, the number of aspects included in the video. ^***^Indicates a highly significant correlation, ^**^indicates a significant correlation, and ^*^indicates a weakly significant correlation.

Pearson correlation analysis showed a positive correlation between video length and the number of saves (r = 0.212, *p* = 0.003). Additionally, a positive correlation was observed between video length and the overall video quality score (r = 0.274, *p* < 0.001), actionability (r = 0.250, *p* < 0.001), and overall quality (r = 0.381, *p* < 0.001). Regarding user engagement, a positive correlation existed between the number of video likes and understandability (r = 0.172, *p* = 0.015). Furthermore, positive correlations were identified between the number of video likes and the number of comments (r = 0.495, *p* < 0.001), saves (r = 0.797, *p* < 0.001), and shares (r = 0.626, *p* < 0.001) (see Figure 5).

## 4 Discussion

Since its introduction in September 2016, TikTok has garnered over 1 billion monthly active users globally, thereby positioning itself as the fastest-growing social media platform (29). The videos selected for this study received approximately 450,000 likes and 40,000 comments roughly 1 year after being posted. This underscores the considerable potential of TikTok as a platform for information dissemination, making it an excellent avenue for health communication and enhancing patient education through short video formats. Individuals with chronic eye conditions, such as DE, need routine ophthalmic evaluations and suitable medical treatment to effectively manage and reduce the long-term impacts of these conditions on their eye health and vision (30). Previous studies have evaluated the quality of some videos on TikTok related to diabetes (31), chronic obstructive pulmonary disease, and other conditions (32–34). However, there is limited focus on TikTok videos concerning DE domestically.

In this study, medical professionals contributed the majority of the videos (81%); however, the total counts of likes, saves, and shares they received was significantly lower compared to those from news organizations and non-medical individual accounts. This discrepancy may be related to the inclusion of content in the form of lectures and doctor-patient consultations in outpatient settings (35). Concurrently, our analysis revealed that merely 5.5% of the 199 videos originated from non-medical personal accounts, suggesting that DE may not be widely recognized by the general public.

Therefore, medical professionals should consider expanding their outreach efforts. Simultaneously, they should focus on content presentation, leveraging TikTok’s algorithm and trending tags to enhance video engagement and attract more viewers.

Video uploaders tended to focus on expressing the symptoms of dry eye and how to manage it, rather than its definition and classification. This imbalance in content proportion is also noted in some studies (31, 34, 35). Since management content—particularly regarding medication selection and usage frequency—requires a certain level of expertise, and different types of dry eye necessitate distinct medications and management plans, it is crucial to address these differences. For instance, Cyclosporine A (CsA) has proven effective in relieving the symptoms and signs of evaporative dry eye (36). Therefore, medical professionals must emphasize the importance of proper medication use for DE as well as professional diagnosis by ophthalmologists to reduce the incidence of incorrect medication. Videos should appropriately incorporate content that addresses the often-overlooked aspects of DE definition, classification, and diagnosis, ensuring the public gains a more comprehensive understanding of dry eye-related knowledge.

Consistent with the findings of most other studies (34, 35, 37, 38), this study found that the quality of TikTok videos related to dry eye was moderate to low. Low scores in reliability and treatment options suggest that video uploaders rarely cite authoritative reports as evidence sources, and clear information sources are essential for ensuring reliability. The quality of video information from different sources also varies (35); videos published by news organizations and non-profit organizations tend to exhibit higher reliability and overall quality. In contrast, Videos produced by for-profit organizations obtained the lowest scores. Content from news organizations and non-profit institutions often includes educational contributions from relevant ophthalmologists, yet it is more reliable than content from individual medical practitioners. Therefore, individual medical professionals should verify information before dissemination and consider potential risks or side effects when advising the public. Additionally, the review team at TikTok should strengthen their oversight by enhancing the visibility of high-quality content while reducing the reach of low-quality videos, potentially leading to their removal. This approach will ensure that audiences are presented with high-quality information.

Previously reported error rates for TikTok content dissemination have ranged from 10.6% to 77.8% (35); however, the error rate in this study was only 2%. Several factors may contribute to this lower error rate. Firstly, 90% of the videos are uploaded by ophthalmologists, hospitals, and news organizations, which possess a certain level of authority, thereby reducing the likelihood of spreading misinformation (33). Secondly, in comparison to our reference standards, videos that do not fully articulate risk factors, treatment management, etc., are not categorized as erroneous. Although the information error rate identified in this study is low, there are still potential dangers. Therefore, video uploaders should update their knowledge in accordance with the latest guidelines from authoritative organizations. As they play a key role in disseminating medical knowledge to the public, and given the high engagement with their videos, ensuring accurate information is critical.

These results have practical implications for video content creation and platform operation. The positive correlation between video length and quality suggests to content creators that, while ensuring content quality, appropriately extending video length may help improve user feedback and engagement. However, it is important to note that video length is not the only factor determining video quality; the depth, comprehensibility, and appeal of the content are equally important. Furthermore, a strong positive correlation was observed between the number of video likes and other user engagement indicators (comments, saves, and shares) offers insights for the operation of video platforms. Platforms can optimize recommendation algorithms based on these metrics to increase the exposure of high-quality videos, thereby promoting user engagement and content dissemination. At the same time, platforms should focus on video comprehensibility and encourage creators to produce more easily understandable content.

While this study provides an evaluation of video quality on TikTok, it is important to acknowledge several limitations. Firstly, the selection of videos was based on TikTok’s default listing, which may be subject to algorithmic biases. Secondly, as a cross-sectional study, the findings are limited to the specific timeframe of data collection and do not allow for causal inferences; these findings may evolve over time. Thirdly, the absence of demographic data regarding video viewers limits the understanding of the audience scope for dry eye health promotion. Lastly, the study’s focus on Chinese-language videos, to the exclusion of English and other languages, may impact the generalizability of the results.

## 5 Conclusion

This research evaluated the quality of educational videos about dry eye care on TikTok, finding they attract significant attention but often lack comprehensive coverage. The videos primarily focus on dry eye symptoms, risk factors, and management, neglecting areas like definition and diagnosis. While TikTok videos generally provide reliable content on dry eye, there is a risk of encountering incomplete or inaccurate information. Therefore, the public should exercise caution when seeking information on dry eye syndrome via TikTok, as TikTok cannot replace professional medical advice. To ensure the accuracy of information, video creators should adhere to guidelines or consensus statements issued by authoritative bodies. Additionally, TikTok should enhance its content review processes and encourage creators to produce more accessible and detailed content.

## Data Availability

The original contributions presented in the study are included in the article/Supplementary material, further inquiries can be directed to the corresponding authors.
