# Supplementary material for: Assessing the quality of educational short videos on dry eye care: a cross-sectional study
Source: Front Public Health. 2025 Apr 9;13:1542278. doi: 10.3389/fpubh.2025.1542278 (PMC12014691; doi:10.3389/fpubh.2025.1542278)
Supplement: Supplementary file 1 [file Table_1.docx]

| **Category** | **Criteria** | **Examples** |
| --- | --- | --- |
| **Definition** |  | Dry eye is a multifactorial disease characterized by unstable tear film causing a variety of symptoms and/or visual impairment, potentially accompanied by ocular surface damage. |
| **Signs/Symptoms** | visual symptoms;  Ocular signs | Eye strain;  foreign body sensation;  dry eyes;  eye discomfort;  pain;  redness;  etc |
| **Risk Factors** | Environmental factors;  personal habits;  surgical factors;  others | Inflammation;  increased tear osmolality;  dry environment;wind;  meibomian gland dysfunction;  aging;  wearing contact lenses;  refractive surgery;  conjunctival laxity;  eyelid laxity;  etc |
| **Classification** | based on the concept of tear film–oriented diagnosis | aqueous-deficient;  decreased wettability;  increased evaporation |
| **Diagnosis** | the combination of symptoms and unstable tear film | Dry eye symptoms:  Ocular Surface Disease Index (OSDI);  Mc Monnies questionnaire, Women's Health Study Questionnaire or the dry eye-related QOL score (DEQS)+Decreased TBUT  (unstable tear film) |
| **Treatment** | Progression Control;  intervention;  drug treatment | Artificial tears or hyaluronic acid eye drops;  Tear secretagogues;  Anti-inflammatory treatment;  eyelid hygiene;  Small amounts of lipid or lipid-containing eye drops;  Eye warming;  increasing the room humidity;  etc |

Supplementary Table Reference standard for dry eye disease.
